# Kctd12 and Ulk2 Partner to Regulate Dendritogenesis and Behavior in the Habenular Nuclei

**DOI:** 10.1371/journal.pone.0110280

**Published:** 2014-10-20

**Authors:** Stacey Lee, Patrick Page-McCaw, Joshua T. Gamse

**Affiliations:** 1 Vanderbilt University, Department of Biological Sciences, Nashville, Tennessee, United States of America; 2 Vanderbilt University Medical Center, Department of Molecular Physiology and Biophysics, Nashville, Tennessee, United States of America; Institute of Molecular and Cell Biology, Singapore

## Abstract

The habenular nuclei of the limbic system regulate responses, such as anxiety, to aversive stimuli in the environment. The habenulae receive inputs from the telencephalon via elaborate dendrites that form in the center of the nuclei. The kinase Ulk2 positively regulates dendritogenesis on habenular neurons, and in turn is negatively regulated by the cytoplasmic protein Kctd12. Given that the habenulae are a nexus in the aversive response circuit, we suspected that incomplete habenular dendritogenesis would have profound implications for behavior. We find that Ulk2, which interacts with Kctd12 proteins via a small proline-serine rich domain, promotes branching and elaboration of dendrites. Loss of Kctd12 results in increased branching/elaboration and decreased anxiety. We conclude that fine-tuning of habenular dendritogenesis during development is essential for appropriate behavioral responses to negative stimuli.

## Introduction

Appropriate neuronal morphogenesis is essential for forming the distinct functional domains of each of the hundreds of types of neurons in the brain. Generating the correct size and shape of dendrites is essential for a neuron to satisfactorily sample and process the signals that converge on its dendritic field. Abnormal formation of dendrites is observed in cortical neurons of patients with disorders including Rhett’s syndrome and fragile X syndrome, correlating with the emergence of behavioral symptoms [1]. Dendrite morphogenesis is a multi-step process, which includes growth, branching, and pruning of neuronal processes [2]. Understanding the control of neuronal circuit development is key to understanding normal and abnormal brain function and behavior.

The zebrafish is an excellent system for studying vertebrate dendritogenesis *in vivo*. The embryos are transparent, making them useful for imaging, and are easily manipulated genetically. Relevance for understanding the human brain is high, as there is extensive conservation of neuroanatomical features and gene expression in many regions of the vertebrate brain [3]. Zebrafish are also an amenable model organism for studying how molecular and genetic effects on development ultimately change behavioral outputs [4]–[6].

In particular, the dorsal habenular nuclei are an advantageous region to study dendritogenesis in the central nervous system. The habenular nuclei have a superficial location in the zebrafish brain and have a stereotypical unipolar morphology, which simplifies analysis [7]. The nuclei act as a relay connecting forebrain regions to the dopaminergic and serotonergic networks in the brain [3], [8]. The habenula receives sensory inputs from the pallium, eminentia thalami, and the posterior tuberculum, and sends efferent connections to downstream circuitry via the interpeduncular nucleus and raphe [9]. Although the formation of the habenular nuclei is poorly understood, these structures are highly conserved throughout vertebrate evolution and coordinate cognitive processes including learning, fear response, addiction, and anxiety [3], [8]. Under normal, non-disease conditions, habenular activation is seen in humans when receiving negative feedback from an external source, and in non-human primates when experiencing an aversive stimulus [10], [11]. Overall, the function of the habenula is to process aversive information from the environment and coordinate an appropriate behavioral response.

Malfunction of the habenular circuitry is observed in schizophrenia and depression [12]–[15]. In schizophrenic patients, the habenula is not activated when negative feedback is received from a short-term memory task, but it is activated in control subjects [15]. This indicates a potential mechanism for the impairment of negative feedback learning in schizophrenic patients. Additionally, an increased amount of habenular calcification has been observed in patients with schizophrenia, although the functional significance of this phenomenon is not well studied [16]. There is evidence in both animal models and in patients that the habenula is involved in depression. Congenitally helpless rats exhibit increased metabolic activity in the habenulo-interpeduncular circuit [17]. In patients with major depression, postmortem human studies have shown decreased habenular volume in both the medial and lateral subnuclei [12], [13]. An increase in neuronal activity in the habenula is seen in depressive patients [14]. Furthermore, deep brain stimulation in the habenula has successfully been used to treat a patient with major depression who was unresponsive to traditional pharmacological treatments [18].

Kctd12 genes negatively regulate habenular dendritogenesis [19]. Expression of Kctd12 in the habenular nuclei is conserved throughout the vertebrate lineage [20], [21]. In zebrafish there are two orthologous genes that have distinct patterns of expression in the subnuclei of the habenula. Kctd12.1 is expressed in the lateral subnuclei, while Kctd12.2 is expressed in the medial subnuclei [3], [8], [20].

Kctd12 proteins are found in the cytoplasm of habenular neurons, including within the dendrites [19]. During dendritogenesis, Kctd12 regulates the activity of Ulk2, a serine/threonine kinase that promotes filopodial extension during neuronal process formation [22], [23]. Ulk2 is expressed throughout both habenular subnuclei, beginning at 48 hours post fertilization (hpf) until at least 96 hpf [19]. Previous work has shown that Kctd12.1 interacts with Ulk2. The presence of Kctd12 appears to inhibit Ulk2 activity, and as a result, dendritogenesis is reduced [19]. Daam1, a formin family protein, also mediates habenular dendritogenesis as well as axiogenesis [24]. However, the mechanisms by which Kctd12 inhibits Ulk2 activity to modulate habenular dendritogenesis are not known.

In this study we focus on biochemical and genetic mechanisms of dendrite formation by Kctd12 and Ulk2 in the habenulae, and the functional repercussions on behavior when dendritogenesis is altered. We expound upon the previously reported Kctd12-Ulk2 regulation of habenular dendritogenesis and show that Kctd12 and Ulk2 interact biochemically, regulate arborization of habenular dendrites, and ultimately affect behavior. Specifically, we show that Kctd12.1 interacts with a 26-amino acid segment of the PS domain of Ulk2. We then demonstrate that Ulk2 promotes branching and elaboration of developing dendrites, but has no effect on extension or retraction events. Finally, we show that increased habenular dendritogenesis decreases anxiety-like behavior. We conclude that Kctd12/Ulk2 regulates the development and ultimately the function of the habenular nuclei.

## Methods

### Zebrafish maintenance and strains

Zebrafish (*Danio rerio*) embryos were obtained by natural spawning and raised at 28.5°C on a 14∶10 light/dark cycle. Staging was by age (dpf; days post-fertilization). To prevent pigment formation, 0.003% phenylthiourea was added to embryo media during development. Zebrafish lines used: Tg[*cfos:gal4vp16*]^s1^°^19t^ (Hb:Gal4 hereafter), *kctd12.1* ^vu442^, and *kctd12.2* ^fh312^ [19], [25]. Zebrafish adults and embryos were euthanized with an overdose of Tricaine applied in the water. The Vanderbilt University Institutional Animal Care and Use Committee approved all animal work (protocol number C/07/024).

### Cloning

The Ulk2 PS domain was divided into four fragments encoding 129–131 amino acids each and these fragments were cloned either individually or in combination into the pGBK bait vector. Fragments correspond to the following amino acids of Ulk2: PS-1 (272−401), PS-2 (401−531), PS-3 (531−61), PS-4 (661−790). These fragments were tested for interaction with Kctd12.1 in a yeast 2-hybrid assay (Matchmaker Gold Yeast 2-Hybrid System, Clontech). Subsequently, the first fragment (PS-1) was divided into five smaller fragments of 26 amino acids. Fragments correspond to the following amino acids of Ulk2: PS1.1 (272−297), PS1.2 (298−323), PS1.3 (324−349), PS1.4 (350−375), PS1.5 (376−401). Adjacent segments were cloned into the pGBK vector. These fragments were tested again by yeast 2-hybrid.

### Yeast 2-hybrid

Kctd12.1 was cloned into an activation domain fusion protein plasmid (pGAD), and Ulk2 fragments into a DNA-binding plasmid (pGBK). Y2H Gold (Clontech) yeast were cotransformed according to the manufacturer’s instructions with pGAD and pGBK fusion plasmids. Yeast were grown on synthetic media lacking leucine and tryptophan (−LEU, −TRP) to select for plasmid uptake. Single colonies were inoculated in liquid media lacking leucine and tryptophan, adjusted to and OD_600_ of 1, and five fold serial dilutions were prepared in sterile water. Five microliters of each dilution were spotted in parallel on –LEU –TRP plates and on plates additionally lacking adenine and histidine (−ADE, −HIS, −LEU, −TRP). Growth was monitored after 2–3 days incubation at 30°C.

### Morpholino knockdown of Ulk2

Morpholino antisense oligonucleotides (Gene Tools) were injected into the yolk underneath the blastomere(s) of 1–2 cell stage embryos (ulk2^MO^). The splice site morpholino was injected at 2 ng/embryo, resulting in a ∼50% knockdown of protein, as previously described [19]. Wild-type sibling controls were not injected with morpholino.

### Transgenesis

Transient transgenic animals were generated using transgenes constructed with the Tol2kit [26]. Transgenic Hb:Gal4 embryos were injected between 2–8 cell stage with a Tol2 construct containing the upstream activating sequence (UAS) driving expression of GFP fused to a CaaX motif (UAS:CaaXGFP:pA). Embryos were screened for cardiac GFP at 2 days post fertilization, and imaged between days 3–4 or fixed at day 4.

### Immunofluorescence

Embryos for whole mount immunohistochemistry were fixed at 4 dpf in Prefer fixative (Anatech) and processed as previously described [19]. Primary antibodies were used at the following concentrations: mouse anti-acetylated tubulin (Sigma-Aldrich) (1∶500) and rabbit anti-GFP (Torrey Pines Biolabs) (1∶500). Primary antibodies were detected using donkey anti-rabbit or goat anti-mouse secondary antibodies conjugated to Alexa 488 or Alexa 568 fluorophores (Invitrogen) (1∶300).

### Microscopy and image analysis

Embryos were anesthetized with Tricaine (Sigma), mounted in 0.6% agarose, and imaged for one hour on a LSM510 META (Zeiss) confocal microscope with a 40X/1.30 Plan NEOFLUAR oil-immersion objective. Z-stacks of the habenula were taken at 1 µm intervals for a total depth of 82 µm. Embryos were maintained at 28.5°C using a forced air heating chamber. Whole mount embryos were imaged in the same manner. Images were processed using Volocity (Improvision) software. Branches of the single dendritic protrusion from habenular neurons [7] were counted manually. A protrusion was counted as a branch (rather than a varicosity) if it was at least 0.70 µm long. Dendrite length was calculated using Simple Neurite Tracer (ImageJ). A total of 38 sibling larvae were quantified, with 21 WT larvae and 17 morphant larvae measured. Fifty-seven neurons were quantified in WT larvae, and forty-five in morphant larvae. Individual dendrites during time-lapse imaging were quantified by on how many extension and retraction events occurred during the imaging period. Sixteen WT and sixteen morphant dendrites were quantified from 4 WT and 4 morphant larvae. Only dendrites that could be unambiguously tracked were selected for quantification. An extension was counted as the protrusion lengthening, and a retraction as the protrusion shortening.

### Behavior

Behavioral assays were performed in a 6-well dish on 5 dpf larvae. Larvae were acclimated on a clear background for 2 minutes, and then placed on a half clear/half black background for the choice portion of the experiment. Images were taken every half second for 10 minutes. Images were processed using FIJI to determine the xy position of the fish in all movie frames [27]. Position of the fish was converted to polar coordinates using the R software environment (r-project.org) and the well was divided into clear and dark background halves. Each half was then subdivided by the distance from the center of the well into the 60% of the area at the center and 40% at the edge. We observed highly variable preference for the center v. edge on the light half, while preference for the center for the edge was consistent between experiments. Total time in the arena does not always equal 1, as counts are normalized to total number of frames, not total number of observations.

### Statistics

Statistical analysis consisted of one-way ANOVAs using web-based software at http://vassarstats.net/anova1 u.html.

## Results and Discussion

### Kctd12 interacts with Ulk2 via a 26-amino acid sequence in the PS domain

**Figure 1:**
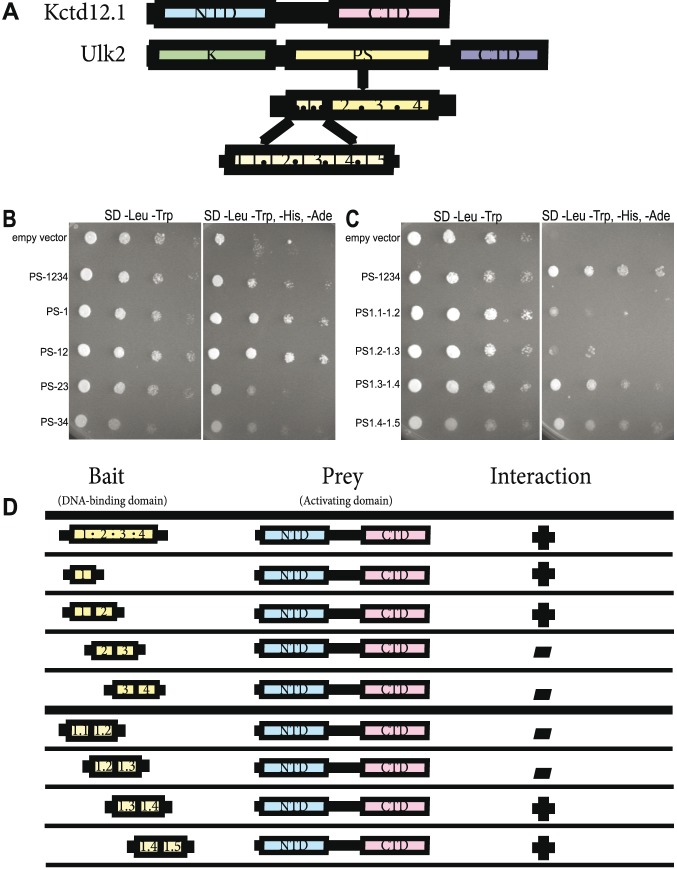
Kctd12.1 interacts with a subset of amino acids in the PS domain of Ulk2. Transformants expressing a fragment of the PS domain of Ulk2 fused to the Gal4 DNA-binding domain were mated with transformants expressing Kctd12.1 fused to the Gal4 activation domain. **A.** Kctd12.1 contains two domains: an N-terminal domain (NTD) that promotes oligomerization, and a C-terminal domain (CTD) of undefined function. Ulk2 contains three domains: an N-terminal serine-threonine kinase domain (K), an internal proline-serine-rich region (PS rich), and a CTD involved in protein–protein interactions. Fragment 1.4 of the Ulk2 PS rich domain is the site of interaction with Kctd12. **B.** Region 1 of the Ulk2 PS domain is the site of interaction with Kctd12.1. **C.** PS domain fragments containing region 1.4 (PS1.3–1.4 and PS1.4–1.5) interact most strongly with Kctd12.1, suggesting the site of interaction is PS1.4. **D.** Summary of the yeast two-hybrid results. Fragment 1.4 of the Ulk2 PS rich domain is the site of interaction with Kctd12.

Previous work showed that Kctd12.1 and Ulk2 interact, which may be important for the regulation of Ulk2 activity [19]. Ulk2 protein contains three distinct domains: an N-terminal kinase domain, a carboxy-terminal domain, and a highly disordered proline and serine-rich middle domain (PS domain) (Figure 1A). The exact function of the PS domain is poorly understood; however, it is the only domain required for interaction with Kctd12.1 [19]. The PS domain has been shown as a site of autophosphorylation that is essential for Ulk2 activation [22]. We sought to identify parts of the PS domain that are required for interaction with Kctd12.1 using the yeast 2-hybrid assay. The PS domain (519 amino acids) was divided into 4 subdomains (each containing 129–131 amino acids) and those regions were expressed either individually or in combination as indicated (Figure 1A). The first subdomain (PS-1) was sufficient for interaction with Kctd12.1 (Figure 1B, D). We further narrowed down the potential interaction site by subdividing the PS-1 into five equal segments (each containing 26 amino acids), and expressing constructs containing overlapping combinations of these segments. This analysis showed that an interaction with Kctd12.1 only occurred when segment 1.4 was included in the assay (Figure 1C, D). We concluded that segment 1.4 within the PS domain of Ulk2 is required for interaction with Kctd12.1.

Since Ulk2 autophosphorylation is required for activity, we hypothesize that Kctd12 binds to segment 1.4 of the PS domain to prevent autophosphorylation and thereby negatively regulate Ulk2 function. Segment 1.4 contains 2 serines and 1 threonine. Future studies will indicate whether one or more of these residues are autophosphorylated, and whether or not Kctd12 can inhibit Ulk2 autophosphorylation in the absence of these key amino acids. It is also possible that other residues outside segment 1.4 are phosphorylated, and the inhibition of phosphorylation is indirect. It will also be interesting to see the phenotypic effect of a Kctd12-insensitive Ulk2 variant *in vivo*.

### Ulk2 positively regulates dendritic branch formation

**Figure 2:**
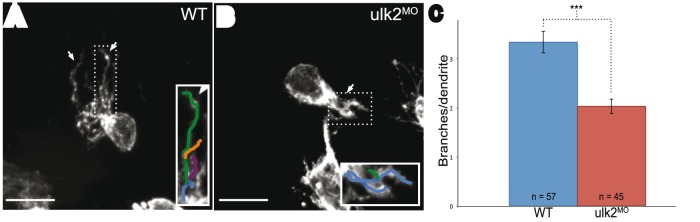
Ulk2 promotes dendritic branching. **A and B.** Habenular neurons were scatter labeled using Hb:Gal4 and UAS:CaaXGFP to label isolated neurons. Arrows point to dendrites on WT (highly branched) and morphant (reduced branched) neurons. Insets demonstrate how branches were quantified; each branch of a dendrite is drawn in a different color. Scale bar is 10 µm. C. Branches per dendrite were calculated by dividing the total number of branches by the number of dendrites per larva. Fifty-seven neurons were quantified in WT larvae and forty-five in morphant larvae. Ulk2 morphants had a decreased number of branches relative to WT (***p<0.0001, ANOVA).

Previously we reported a decrease in neuropil volume in Ulk2 morphants, but the underlying mechanism of this phenomenon is not understood [19]. Ulk2 could potentially alter neuropil volume by affecting dendrite extension, retraction, or branching. To test the specific function of Ulk2 on habenular dendritogenesis, we used an antisense morpholino oligonucleotide (MO) to interfere with Ulk2 function, and measured dendrite length and branch number at 4 dpf, after most habenular dendritogenesis has been completed. Individual habenular neurons were labeled with membrane-tethered GFP to visualize the dendrites (Figure 2A, B). When compared to controls, we found no apparent change in individual dendrite length (data not shown), but a significant decrease in the number of dendrite branches in Ulk2 morphants (Figure 2C).

Ulk2 has been previously reported to promote early endosome formation and axonal growth, and to suppress axon branching [23], [28]. In contrast, we found that Ulk2 promotes dendritic branching or branch stabilization. Knockdown of Ulk2 protein decreased branch number, without altering branch length. These results indicate that Ulk2 possibly has differing roles in axons and dendrites: in axons it promotes elongation and suppresses branching, while in dendrites it only promotes branching. This phenotype might also be due to cell-type or species differences.

### Ulk2 positively regulates dendrite elaboration

**Figure 3:**
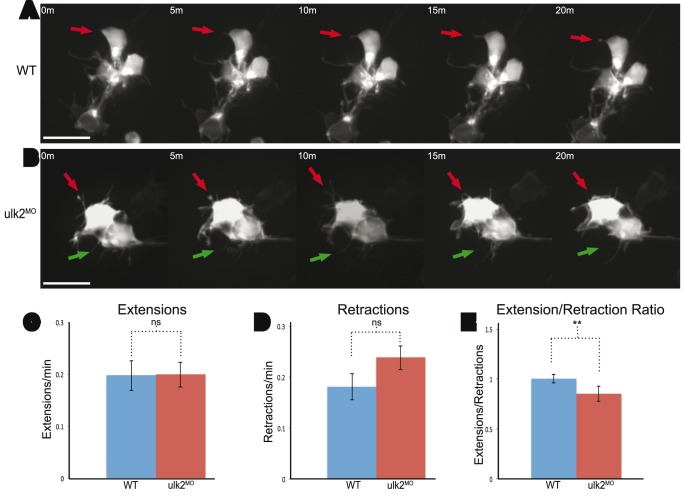
Ulk2 morphants have a decreased extension/retraction ratio. **A. and B.** Habenular neurons were scatter labeled and imaged for one hour. Red arrows point to dendrites being tracked over the 20-minute period displayed. Green arrows point to an additional dendrite tracked during the same time-lapse. Dendrites in the WT neurons are maintained for longer than in Ulk2 morphants. Scale bar is 10 µm. **C. and D.** The number of extension (p = 1) and retraction (p = 0.1095) events per dendrite per minute was similar between WT (n = 16) and Ulk2 morphants (n = 16), but **E.** the ratio of extension events to retraction events per dendrite was significantly reduced (**p = 0.00488, ANOVA) in Ulk2 morphants.

Since fixed embryos can only offer static information on dendritogenesis, we also examined dendrite dynamics by time-lapse imaging. We quantified the number of extensions and retractions of the GFP-labeled dendrites during a one-hour imaging period and compared Ulk2 morphants and controls (Figure 3A, B). We found that there was no difference in the number of extensions or retraction events (Figure 3C, D). However the extension/retraction ratio was reduced in Ulk2 morphants (Figure 3E), which is consistent with our results in fixed embryos and suggests that Ulk2 plays a role in maintaining dendrite elaborations.

Previous work in our lab showed a dramatic decrease in overall dendritic volume [19] when Ulk2 is knocked down, but we had no explanation for the mechanism of this phenotype. While static images are convenient for observing global phenotypic changes in dendrites, time-lapse imaging gave us insight into the dynamics of dendritogenesis. From our time-lapse imaging, we concluded that Ulk2 plays a role in dendrite elaboration. Although Ulk2 knockdown did not change the number of extensions or retractions, the ratio of extensions to retractions decreased in Ulk2 morphants. We did not see a change in cell morphology or polarity. This observation suggests that Ulk2 is required for dendrite elaboration, and without it, dendrites are unable to be maintained and become stable.

### Kctd12 proteins negatively regulate thigmotactic behavior in a non-preferred environment

**Figure 4:**
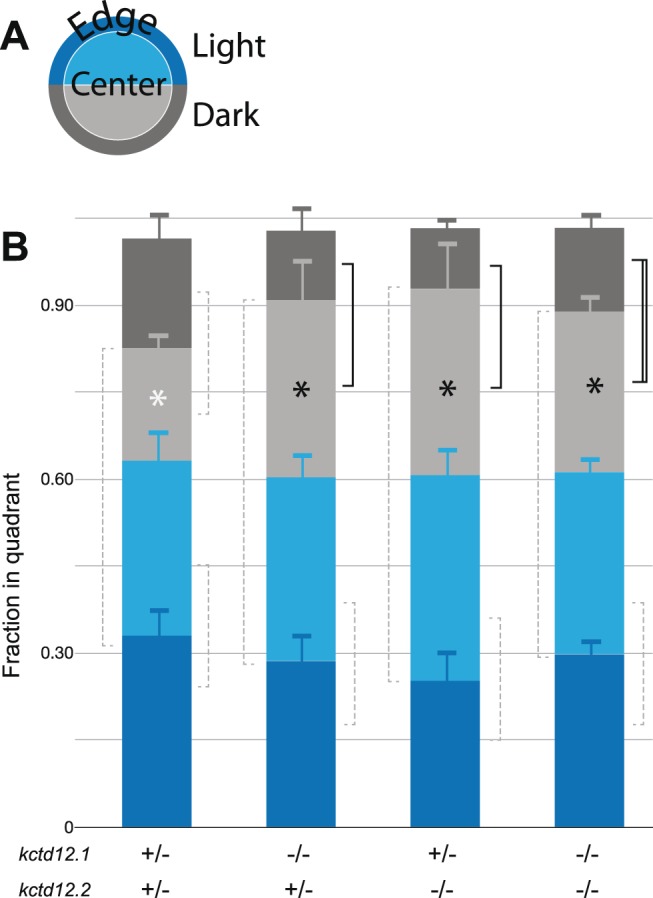
Kctd12 mutation causes a decrease in thigmotactic behavior. **A.** Each well of a 6-well plate is divided into halves with dark (gray) or clear (blue) bottoms. The well is further divided into center (light) and edge (dark). The position of the fish is recorded every half second to measure scototaxis or thigmotaxis. The center is defined as 60% of the area of the circle (inner) and the edge as the remaining 40% (outer). Double heterozygous larvae were used as controls **B.** Scototaxis. No change in scototaxis is detected between genotypes. All genotypes prefer the light. Dotted lines are ns. Thigmotaxis. Preference for the center increases in Kctd12 single and double mutants (solid line: p<0.05, double line: p<0.01, 2-tailed t-test). Pooling mutant animals for a comparison to double heterozygote controls showed a significant effect of genotype (white vs. black * p<0.01). kctd12.1/2+/− n = 19; kctd12.1/2−/− n = 9; kctd12.1−/−, kctd12.2+/− n = 11; kctd12.1+/−, kctd12.2−/− n = 7.

The habenular nuclei function as an important regulator of various behaviors such as learning, fear response, addiction, escape, and anxiety [8]. Recently, the zebrafish has become a useful model organism to study genetic mechanisms of fear and anxiety related behaviors using paradigms such as scototaxis (dark environmental preference) and thigmotaxis (edge preference) [29], [30]. We investigated the functions of Kctd12 proteins in scototactic and thigmotactic behaviors. We observed no scototactic phenotype, as all genotypes preferred the light, regardless of their genotypes (Figure 4A). However, in the dark, *kctd12* mutants (*kctd12.1*, *kctd12.2*, or double mutants) exhibited an increased preference for the center of the arena, as opposed to the edge (Figure 4B). With these experiments, we cannot rule out that this behavior phenotype is not due to changes in non-habenular neurons where Kctd12 is expressed. Kctd12.1 is also expressed in the retina at 96 hpf [20], [26], [31]. Kctd12.2 is expressed in rhombomere 4 from 10–13 somites and in various small groups of neurons in the forebrain between 48–96 hpf [20], [32]. Although Kctd12 expression is not restricted to the habenula, it is the only place where both Kctd12.1 and 12.2 are expressed together. Since the habenula and thigmotaxis are related to anxiety, and mutation of either Kctd12 reduces thigmotaxis, the most parsimonious explanation is that altered Kctd12 function in the habenula is responsible [8], [30], [33], [34].

Our study is the first to implicate Kctd12 genes in the regulation of anxiety-like behavior. Mutation of Kctd12 causes an overelaboration of dendrites in the habenula, consistent with the negative regulation of Ulk2 by Kctd12 [19]. When Kctd12.1 or 12.2 are absent, the larvae appear less anxious. It is unclear how the overelaboration of dendrites causes a decrease in anxiety-like behavior. Future studies to examine the relationship between dendrite volume and behavioral output will be important to parse out the relationship between genes, circuits, and behavior.
