# Correction: Kctd12 and Ulk2 Partner to Regulate Dendritogenesis and Behavior in the Habenular Nuclei

**DOI:** 10.1371/journal.pone.0117637

**Published:** 2015-02-03

**Authors:** The PLOS ONE Staff

The line weights in Fig. 1 are heavier than intended. The publisher apologizes for the error. Please see the correct Fig. 1 here.

**Figure 1:**
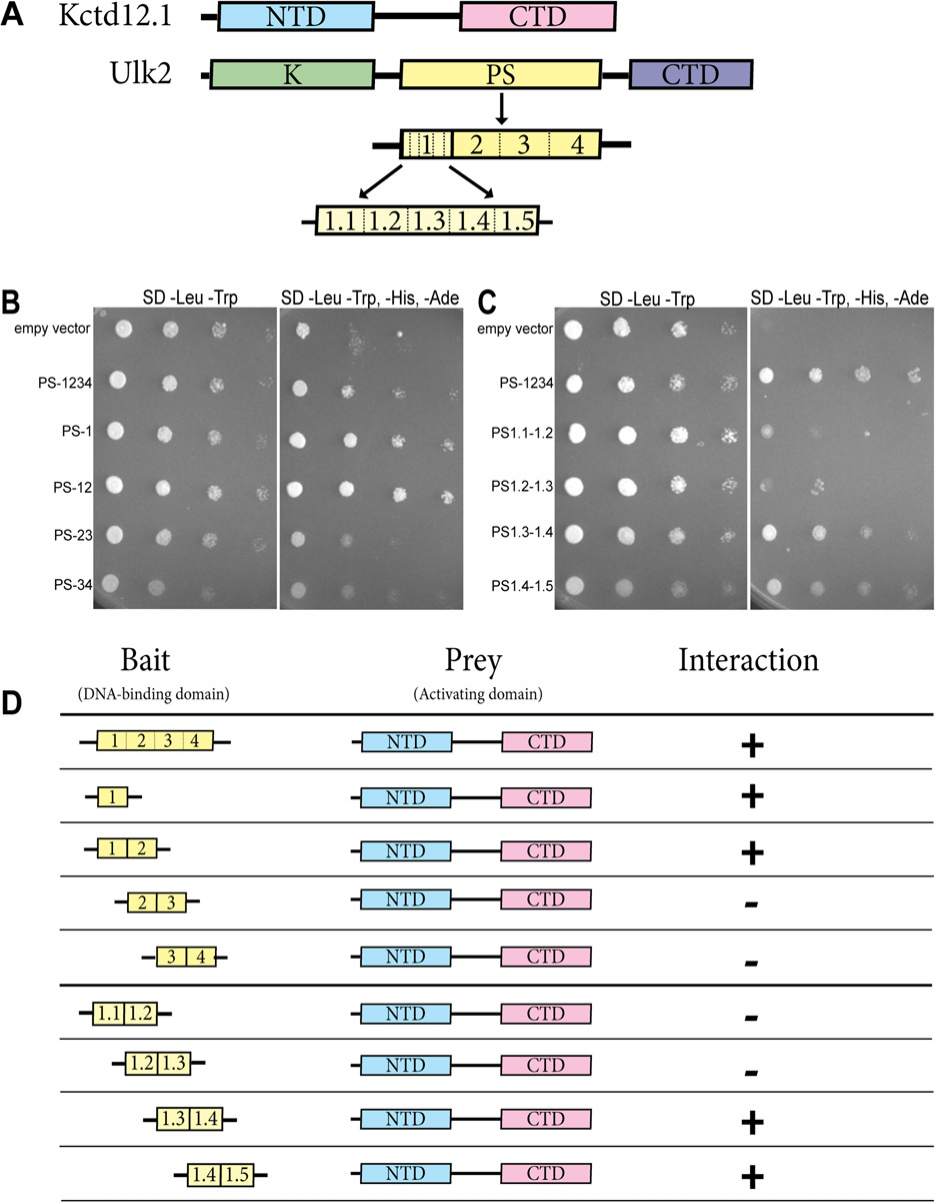
Kctd12.1 interacts with a subset of amino acids in the PS domain of Ulk2. Transformants expressing a fragment of the PS domain of Ulk2 fused to the Gal4 DNA-binding domain were mated with transformants expressing Kctd12.1 fused to the Gal4 activation domain. **A**. Kctd12.1 contains two domains: an N-terminal domain (NTD) that promotes oligomerization, and a C-terminal domain (CTD) of undefined function. Ulk2 contains three domains: an N-terminal serine-threonine kinase domain (K), an internal proline-serine-rich region (PS rich), and a CTD involved in protein–protein interactions. Fragment 1.4 of the Ulk2 PS rich domain is the site of interaction with Kctd12. **B**. Region 1 of the Ulk2 PS domain is the site of interaction with Kctd12.1. **C**. PS domain fragments containing region 1.4 (PS1.3–1.4 and PS1.4–1.5) interact most strongly with Kctd12.1, suggesting the site of interaction is PS1.4. **D**. Summary of the yeast two-hybrid results. Fragment 1.4 of the Ulk2 PS rich domain is the site of interaction with Kctd12.
